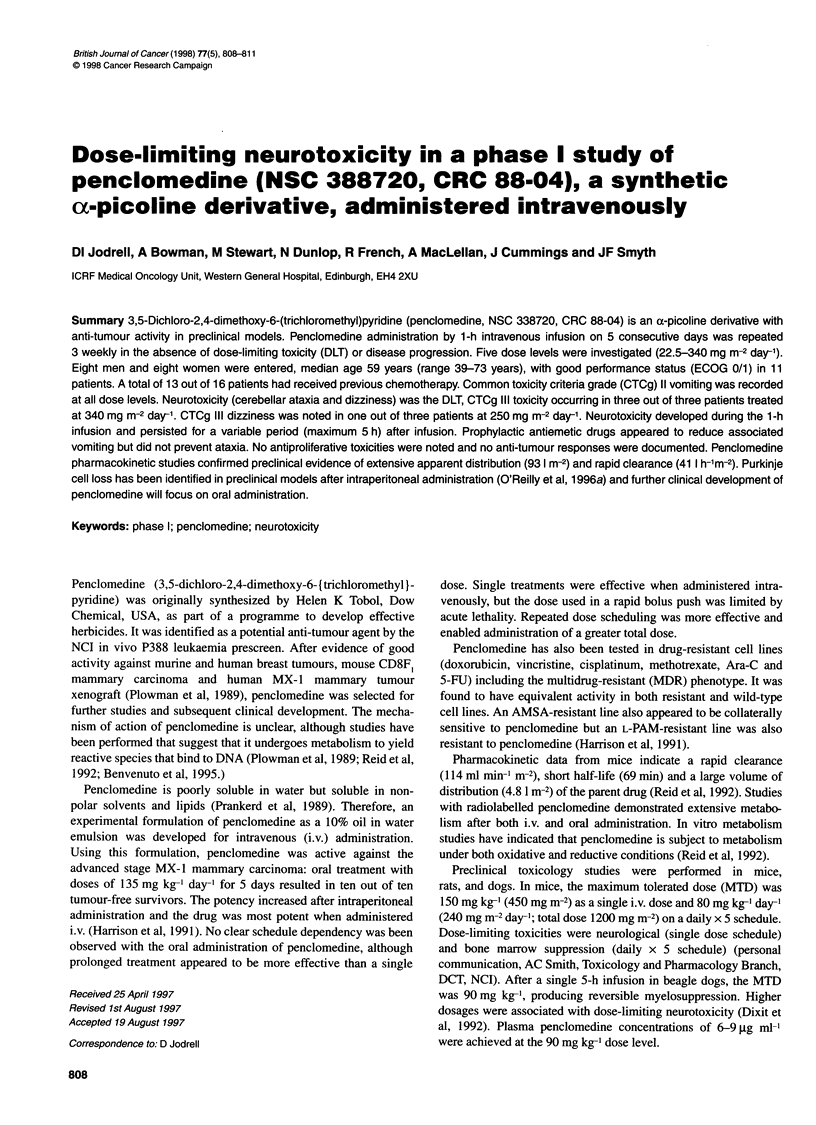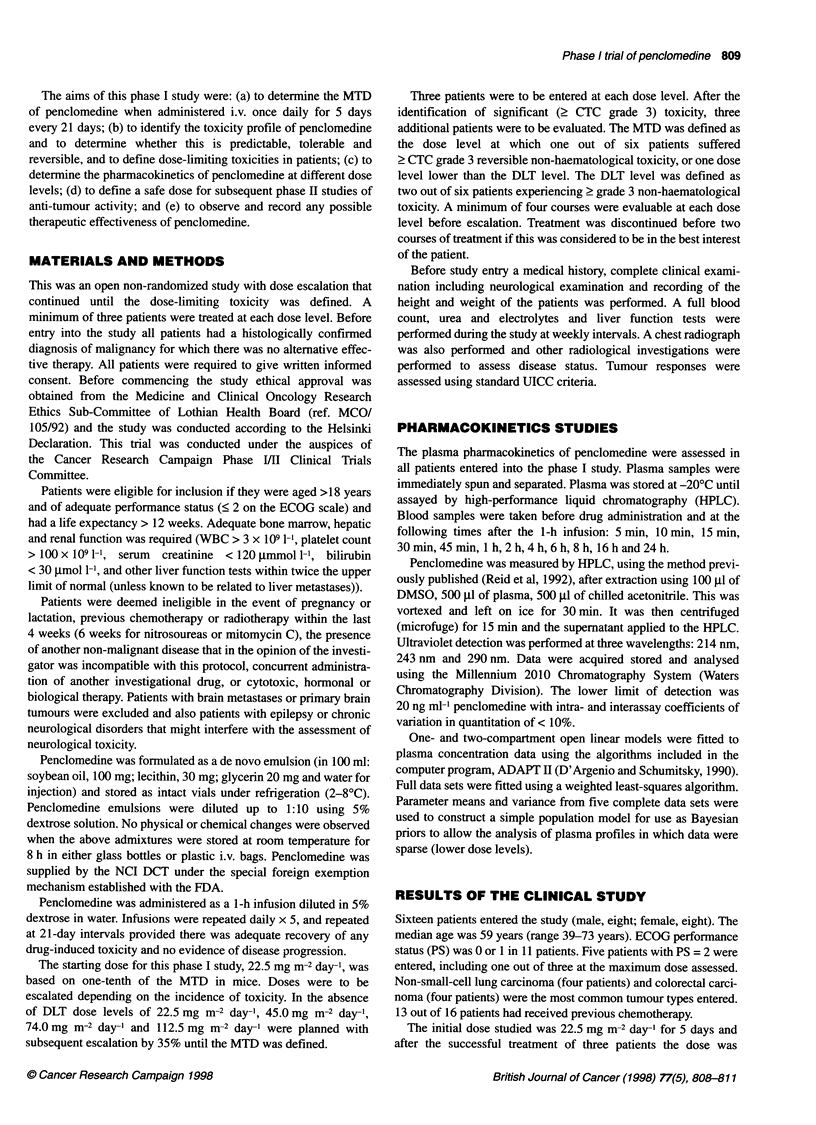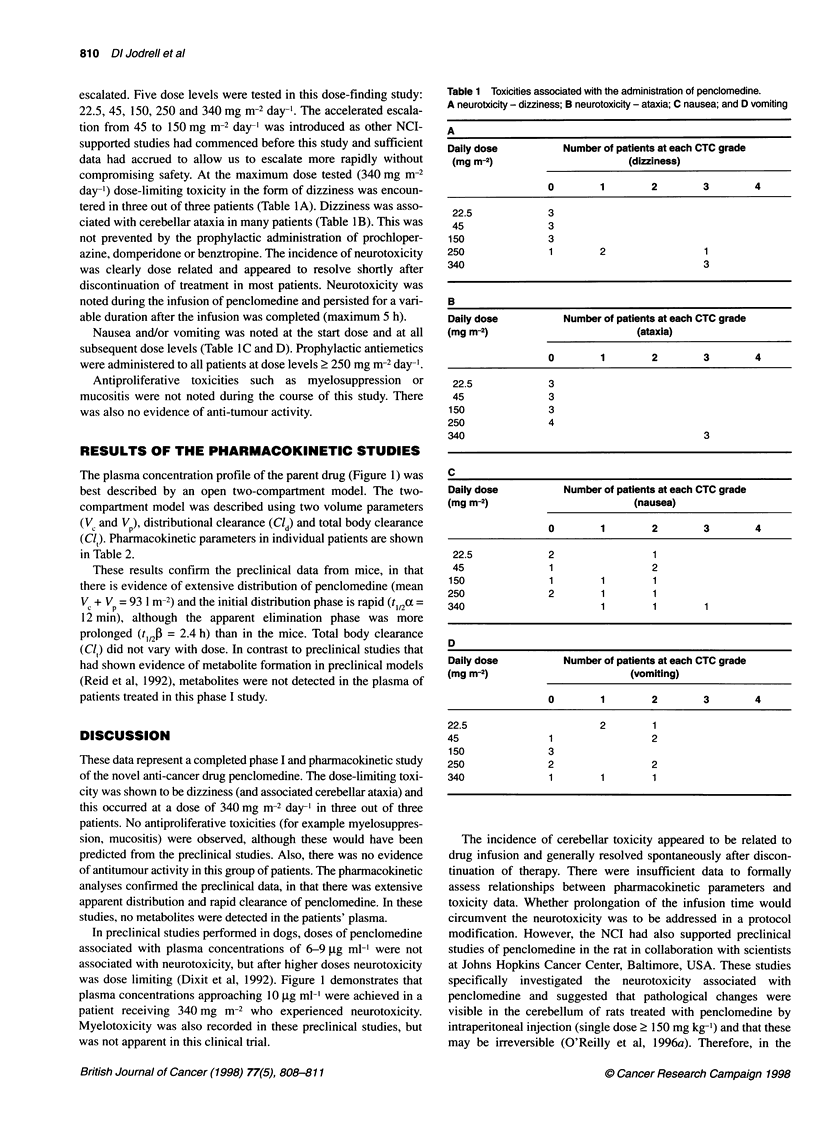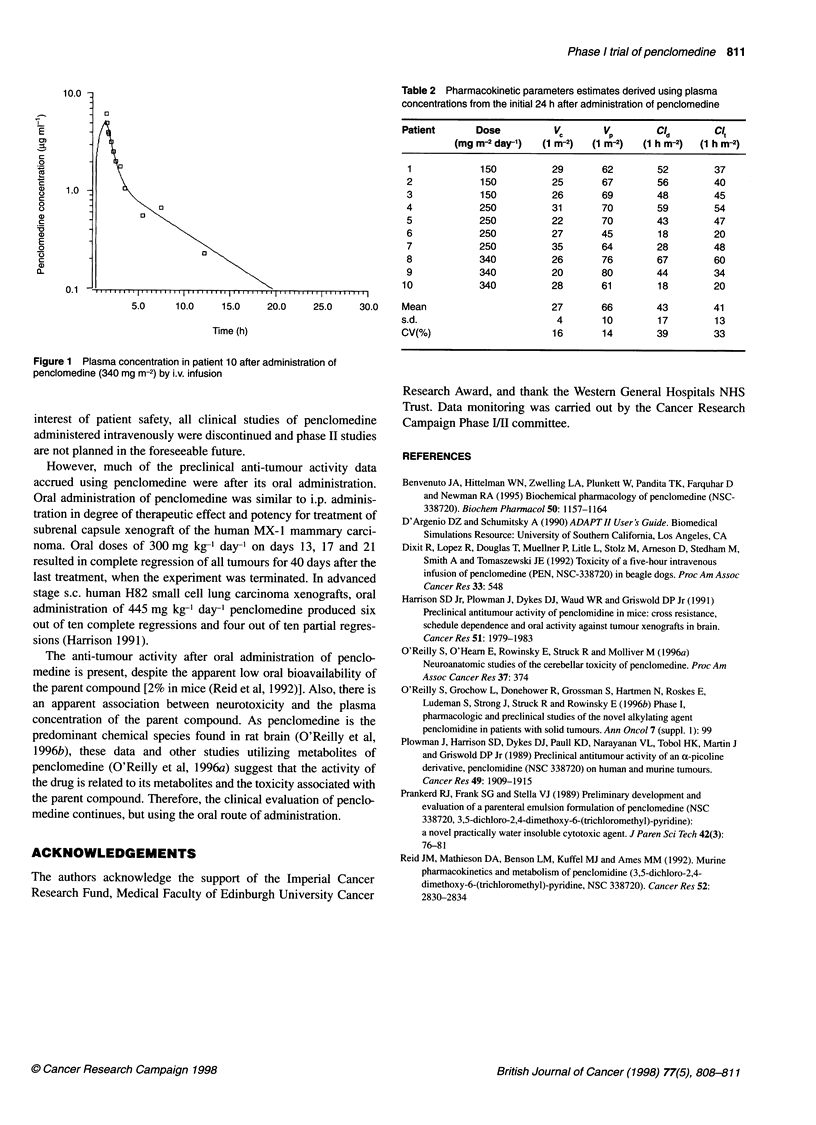# Dose-limiting neurotoxicity in a phase I study of penclomedine (NSC 388720, CRC 88-04), a synthetic alpha-picoline derivative, administered intravenously.

**DOI:** 10.1038/bjc.1998.131

**Published:** 1998-03

**Authors:** D. I. Jodrell, A. Bowman, M. Stewart, N. Dunlop, R. French, A. MacLellan, J. Cummings, J. F. Smyth

**Affiliations:** ICRF Medical Oncology Unit, Western General Hospital, Edinburgh.

## Abstract

3,5-Dichloro-2,4-dimethoxy-6-(trichloromethyl)pyridine (penclomedine, NSC 338720, CRC 88-04) is an alpha-picoline derivative with anti-tumour activity in preclinical models. Penclomedine administration by 1-h intravenous infusion on 5 consecutive days was repeated 3 weekly in the absence of dose-limiting toxicity (DLT) or disease progression. Five dose levels were investigated (22.5-340 mg m(-2) day[-1]). Eight men and eight women were entered, median age 59 years (range 39-73 years), with good performance status (ECOG 0/1) in 11 patients. A total of 13 out of 16 patients had received previous chemotherapy. Common toxicity criteria grade (CTCg) II vomiting was recorded at all dose levels. Neurotoxicity (cerebellar ataxia and dizziness) was the DLT, CTCg III toxicity occurring in three out of three patients treated at 340 mg m(-2) day(-1). CTCg III dizziness was noted in one out of three patients at 250 mg m(-2) day(-1). Neurotoxicity developed during the 1-h infusion and persisted for a variable period (maximum 5 h) after infusion. Prophylactic antiemetic drugs appeared to reduce associated vomiting but did not prevent ataxia. No antiproliferative toxicities were noted and no anti-tumour responses were documented. Penclomedine pharmacokinetic studies confirmed preclinical evidence of extensive apparent distribution (93 l m[-2]) and rapid clearance (41 l h[-1] m[-2]). Purkinje cell loss has been identified in preclinical models after intraperitoneal administration (O'Reilly et al, 1996a) and further clinical development of penclomedine will focus on oral administration.


British Journal of Cancer (1998) 77(5), 808–811
© 1998 Cancer Research Campaign

### Dose-limiting neurotoxicity in a phase I study of penclomedine (NSC 388720, CRC 88-04), a synthetic α-picoline derivative, administered intravenously

DI Jodrell, A Bowman, M Stewart, N Dunlop, R French, A MacLellan, J Cummings and JF Smyth

ICRF Medical Oncology Unit, Western General Hospital, Edinburgh, EH4 2XU

**Summary** 3,5-Dichloro-2,4-dimethoxy-6-(trichloromethyl)pyridine (penclomedine, NSC 338720, CRC 88-04) is an α-picoline derivative with anti-tumour activity in preclinical models. Penclomedine administration by 1-h intravenous infusion on 5 consecutive days was repeated 3 weekly in the absence of dose-limiting toxicity (DLT) or disease progression. Five dose levels were investigated (22.5–340 mg $m^{-2}$ $day^{-1}$). Eight men and eight women were entered, median age 59 years (range 39–73 years), with good performance status (ECOG 0/1) in 11 patients. A total of 13 out of 16 patients had received previous chemotherapy. Common toxicity criteria grade (CTCg) II vomiting was recorded at all dose levels. Neurotoxicity (cerebellar ataxia and dizziness) was the DLT, CTCg III toxicity occurring in three out of three patients treated at 340 mg $m^{-2}$ $day^{-1}$. CTCg III dizziness was noted in one out of three patients at 250 mg $m^{-2}$ $day^{-1}$. Neurotoxicity developed during the 1-h infusion and persisted for a variable period (maximum 5 h) after infusion. Prophylactic antiemetic drugs appeared to reduce associated vomiting but did not prevent ataxia. No antiproliferative toxicities were noted and no anti-tumour responses were documented. Penclomedine pharmacokinetic studies confirmed preclinical evidence of extensive apparent distribution (93 l $m^{-2}$) and rapid clearance (41 l $h^{-1}m^{-2}$). Purkinje cell loss has been identified in preclinical models after intraperitoneal administration (O'Reilly et al, 1996*a*) and further clinical development of penclomedine will focus on oral administration.

**Keywords:** phase I; penclomedine; neurotoxicity

Penclomedine (3,5-dichloro-2,4-dimethoxy-6-{trichloromethyl}-pyridine) was originally synthesized by Helen K Tobol, Dow Chemical, USA, as part of a programme to develop effective herbicides. It was identified as a potential anti-tumour agent by the NCI in vivo P388 leukaemia prescreen. After evidence of good activity against murine and human breast tumours, mouse $CD8F_1$ mammary carcinoma and human MX-1 mammary tumour xenograft (Plowman et al, 1989), penclomedine was selected for further studies and subsequent clinical development. The mechanism of action of penclomedine is unclear, although studies have been performed that suggest that it undergoes metabolism to yield reactive species that bind to DNA (Plowman et al, 1989; Reid et al, 1992; Benvenuto et al, 1995.)

Penclomedine is poorly soluble in water but soluble in non-polar solvents and lipids (Prankerd et al, 1989). Therefore, an experimental formulation of penclomedine as a 10% oil in water emulsion was developed for intravenous (i.v.) administration. Using this formulation, penclomedine was active against the advanced stage MX-1 mammary carcinoma: oral treatment with doses of 135 mg $kg^{-1}$ $day^{-1}$ for 5 days resulted in ten out of ten tumour-free survivors. The potency increased after intraperitoneal administration and the drug was most potent when administered i.v. (Harrison et al, 1991). No clear schedule dependency was been observed with the oral administration of penclomedine, although prolonged treatment appeared to be more effective than a single dose. Single treatments were effective when administered intravenously, but the dose used in a rapid bolus push was limited by acute lethality. Repeated dose scheduling was more effective and enabled administration of a greater total dose.

Penclomedine has also been tested in drug-resistant cell lines (doxorubicin, vincristine, cisplatinum, methotrexate, Ara-C and 5-FU) including the multidrug-resistant (MDR) phenotype. It was found to have equivalent activity in both resistant and wild-type cell lines. An AMSA-resistant line also appeared to be collaterally sensitive to penclomedine but an L-PAM-resistant line was also resistant to penclomedine (Harrison et al, 1991).

Pharmacokinetic data from mice indicate a rapid clearance (114 ml $min^{-1}$ $m^{-2}$), short half-life (69 min) and a large volume of distribution (4.8 l $m^{-2}$) of the parent drug (Reid et al, 1992). Studies with radiolabelled penclomedine demonstrated extensive metabolism after both i.v. and oral administration. In vitro metabolism studies have indicated that penclomedine is subject to metabolism under both oxidative and reductive conditions (Reid et al, 1992).

Preclinical toxicology studies were performed in mice, rats, and dogs. In mice, the maximum tolerated dose (MTD) was 150 mg $kg^{-1}$ (450 mg $m^{-2}$) as a single i.v. dose and 80 mg $kg^{-1}$ $day^{-1}$ (240 mg $m^{-2}$ $day^{-1}$; total dose 1200 mg $m^{-2}$) on a daily × 5 schedule. Dose-limiting toxicities were neurological (single dose schedule) and bone marrow suppression (daily × 5 schedule) (personal communication, AC Smith, Toxicology and Pharmacology Branch, DCT, NCI). After a single 5-h infusion in beagle dogs, the MTD was 90 mg $kg^{-1}$, producing reversible myelosuppression. Higher dosages were associated with dose-limiting neurotoxicity (Dixit et al, 1992). Plasma penclomedine concentrations of 6–9 μg $ml^{-1}$ were achieved at the 90 mg $kg^{-1}$ dose level.

*Received 25 April 1997*
*Revised 1st August 1997*
*Accepted 19 August 1997*

*Correspondence to:* D Jodrell

The aims of this phase I study were: (a) to determine the MTD of penclomedine when administered i.v. once daily for 5 days every 21 days; (b) to identify the toxicity profile of penclomedine and to determine whether this is predictable, tolerable and reversible, and to define dose-limiting toxicities in patients; (c) to determine the pharmacokinetics of penclomedine at different dose levels; (d) to define a safe dose for subsequent phase II studies of anti-tumour activity; and (e) to observe and record any possible therapeutic effectiveness of penclomedine.

### MATERIALS AND METHODS

This was an open non-randomized study with dose escalation that continued until the dose-limiting toxicity was defined. A minimum of three patients were treated at each dose level. Before entry into the study all patients had a histologically confirmed diagnosis of malignancy for which there was no alternative effective therapy. All patients were required to give written informed consent. Before commencing the study ethical approval was obtained from the Medicine and Clinical Oncology Research Ethics Sub-Committee of Lothian Health Board (ref. MCO/105/92) and the study was conducted according to the Helsinki Declaration. This trial was conducted under the auspices of the Cancer Research Campaign Phase I/II Clinical Trials Committee.

Patients were eligible for inclusion if they were aged >18 years and of adequate performance status (≤ 2 on the ECOG scale) and had a life expectancy > 12 weeks. Adequate bone marrow, hepatic and renal function was required (WBC > $3 \times 10^9$ $l^{-1}$, platelet count > $100 \times 10^9$ $l^{-1}$, serum creatinine < 120 μmmol $l^{-1}$, bilirubin < 30 μmol $l^{-1}$, and other liver function tests within twice the upper limit of normal (unless known to be related to liver metastases)).

Patients were deemed ineligible in the event of pregnancy or lactation, previous chemotherapy or radiotherapy within the last 4 weeks (6 weeks for nitrosoureas or mitomycin C), the presence of another non-malignant disease that in the opinion of the investigator was incompatible with this protocol, concurrent administration of another investigational drug, or cytotoxic, hormonal or biological therapy. Patients with brain metastases or primary brain tumours were excluded and also patients with epilepsy or chronic neurological disorders that might interfere with the assessment of neurological toxicity.

Penclomedine was formulated as a de novo emulsion (in 100 ml: soybean oil, 100 mg; lecithin, 30 mg; glycerin 20 mg and water for injection) and stored as intact vials under refrigeration (2–8°C). Penclomedine emulsions were diluted up to 1:10 using 5% dextrose solution. No physical or chemical changes were observed when the above admixtures were stored at room temperature for 8 h in either glass bottles or plastic i.v. bags. Penclomedine was supplied by the NCI DCT under the special foreign exemption mechanism established with the FDA.

Penclomedine was administered as a 1-h infusion diluted in 5% dextrose in water. Infusions were repeated daily × 5, and repeated at 21-day intervals provided there was adequate recovery of any drug-induced toxicity and no evidence of disease progression.

The starting dose for this phase I study, 22.5 mg $m^{-2}$ $day^{-1}$, was based on one-tenth of the MTD in mice. Doses were to be escalated depending on the incidence of toxicity. In the absence of DLT dose levels of 22.5 mg $m^{-2}$ $day^{-1}$, 45.0 mg $m^{-2}$ $day^{-1}$, 74.0 mg $m^{-2}$ $day^{-1}$ and 112.5 mg $m^{-2}$ $day^{-1}$ were planned with subsequent escalation by 35% until the MTD was defined.

Three patients were to be entered at each dose level. After the identification of significant (≥ CTC grade 3) toxicity, three additional patients were to be evaluated. The MTD was defined as the dose level at which one out of six patients suffered ≥ CTC grade 3 reversible non-haematological toxicity, or one dose level lower than the DLT level. The DLT level was defined as two out of six patients experiencing ≥ grade 3 non-haematological toxicity. A minimum of four courses were evaluable at each dose level before escalation. Treatment was discontinued before two courses of treatment if this was considered to be in the best interest of the patient.

Before study entry a medical history, complete clinical examination including neurological examination and recording of the height and weight of the patients was performed. A full blood count, urea and electrolytes and liver function tests were performed during the study at weekly intervals. A chest radiograph was also performed and other radiological investigations were performed to assess disease status. Tumour responses were assessed using standard UICC criteria.

### PHARMACOKINETICS STUDIES

The plasma pharmacokinetics of penclomedine were assessed in all patients entered into the phase I study. Plasma samples were immediately spun and separated. Plasma was stored at –20°C until assayed by high-performance liquid chromatography (HPLC). Blood samples were taken before drug administration and at the following times after the 1-h infusion: 5 min, 10 min, 15 min, 30 min, 45 min, 1 h, 2 h, 4 h, 6 h, 8 h, 16 h and 24 h.

Penclomedine was measured by HPLC, using the method previously published (Reid et al, 1992), after extraction using 100 μl of DMSO, 500 μl of plasma, 500 μl of chilled acetonitrile. This was vortexed and left on ice for 30 min. It was then centrifuged (microfuge) for 15 min and the supernatant applied to the HPLC. Ultraviolet detection was performed at three wavelengths: 214 nm, 243 nm and 290 nm. Data were acquired stored and analysed using the Millennium 2010 Chromatography System (Waters Chromatography Division). The lower limit of detection was 20 ng $ml^{-1}$ penclomedine with intra- and interassay coefficients of variation in quantitation of < 10%.

One- and two-compartment open linear models were fitted to plasma concentration data using the algorithms included in the computer program, ADAPT II (D'Argenio and Schumitsky, 1990). Full data sets were fitted using a weighted least-squares algorithm. Parameter means and variance from five complete data sets were used to construct a simple population model for use as Bayesian priors to allow the analysis of plasma profiles in which data were sparse (lower dose levels).

### RESULTS OF THE CLINICAL STUDY

Sixteen patients entered the study (male, eight; female, eight). The median age was 59 years (range 39–73 years). ECOG performance status (PS) was 0 or 1 in 11 patients. Five patients with PS = 2 were entered, including one out of three at the maximum dose assessed. Non-small-cell lung carcinoma (four patients) and colorectal carcinoma (four patients) were the most common tumour types entered. 13 out of 16 patients had received previous chemotherapy.

The initial dose studied was 22.5 mg $m^{-2}$ $day^{-1}$ for 5 days and after the successful treatment of three patients the dose was

© Cancer Research Campaign 1998

escalated. Five dose levels were tested in this dose-finding study: 22.5, 45, 150, 250 and 340 mg $m^{-2}$ $day^{-1}$. The accelerated escalation from 45 to 150 mg $m^{-2}$ $day^{-1}$ was introduced as other NCI-supported studies had commenced before this study and sufficient data had accrued to allow us to escalate more rapidly without compromising safety. At the maximum dose tested (340 mg $m^{-2}$ $day^{-1}$) dose-limiting toxicity in the form of dizziness was encountered in three out of three patients (Table 1A). Dizziness was associated with cerebellar ataxia in many patients (Table 1B). This was not prevented by the prophylactic administration of prochlorperazine, domperidone or benztropine. The incidence of neurotoxicity was clearly dose related and appeared to resolve shortly after discontinuation of treatment in most patients. Neurotoxicity was noted during the infusion of penclomedine and persisted for a variable duration after the infusion was completed (maximum 5 h).

Nausea and/or vomiting was noted at the start dose and at all subsequent dose levels (Table 1C and D). Prophylactic antiemetics were administered to all patients at dose levels ≥ 250 mg $m^{-2}$ $day^{-1}$.

Antiproliferative toxicities such as myelosuppression or mucositis were not noted during the course of this study. There was also no evidence of anti-tumour activity.

**Table 1** Toxicities associated with the administration of penclomedine. **A** neurotxicity – dizziness; **B** neurotoxicity – ataxia; **C** nausea; and **D** vomiting

**A**

| Daily dose (mg $m^{-2}$) | Number of patients at each CTC grade (dizziness) | | | | |
|---|---|---|---|---|---|
| | 0 | 1 | 2 | 3 | 4 |
| 22.5 | 3 | | | | |
| 45 | 3 | | | | |
| 150 | 3 | | | | |
| 250 | 1 | 2 | | 1 | |
| 340 | | | | 3 | |

**B**

| Daily dose (mg $m^{-2}$) | Number of patients at each CTC grade (ataxia) | | | | |
|---|---|---|---|---|---|
| | 0 | 1 | 2 | 3 | 4 |
| 22.5 | 3 | | | | |
| 45 | 3 | | | | |
| 150 | 3 | | | | |
| 250 | 4 | | | | |
| 340 | | | | 3 | |

**C**

| Daily dose (mg $m^{-2}$) | Number of patients at each CTC grade (nausea) | | | | |
|---|---|---|---|---|---|
| | 0 | 1 | 2 | 3 | 4 |
| 22.5 | 2 | | 1 | | |
| 45 | 1 | | 2 | | |
| 150 | 1 | 1 | 1 | | |
| 250 | 2 | 1 | 1 | | |
| 340 | | 1 | 1 | 1 | |

**D**

| Daily dose (mg $m^{-2}$) | Number of patients at each CTC grade (vomiting) | | | | |
|---|---|---|---|---|---|
| | 0 | 1 | 2 | 3 | 4 |
| 22.5 | | 2 | 1 | | |
| 45 | 1 | | 2 | | |
| 150 | 3 | | | | |
| 250 | 2 | | 2 | | |
| 340 | 1 | 1 | 1 | | |

### RESULTS OF THE PHARMACOKINETIC STUDIES

The plasma concentration profile of the parent drug (Figure 1) was best described by an open two-compartment model. The two-compartment model was described using two volume parameters ($V_c$ and $V_p$), distributional clearance ($Cl_d$) and total body clearance ($Cl_t$). Pharmacokinetic parameters in individual patients are shown in Table 2.

These results confirm the preclinical data from mice, in that there is evidence of extensive distribution of penclomedine (mean $V_c + V_p = 93$ l $m^{-2}$) and the initial distribution phase is rapid ($t_{1/2}\alpha$ = 12 min), although the apparent elimination phase was more prolonged ($t_{1/2}\beta$ = 2.4 h) than in the mice. Total body clearance ($Cl_t$) did not vary with dose. In contrast to preclinical studies that had shown evidence of metabolite formation in preclinical models (Reid et al, 1992), metabolites were not detected in the plasma of patients treated in this phase I study.

### DISCUSSION

These data represent a completed phase I and pharmacokinetic study of the novel anti-cancer drug penclomedine. The dose-limiting toxicity was shown to be dizziness (and associated cerebellar ataxia) and this occurred at a dose of 340 mg $m^{-2}$ $day^{-1}$ in three out of three patients. No antiproliferative toxicities (for example myelosuppression, mucositis) were observed, although these would have been predicted from the preclinical studies. Also, there was no evidence of antitumour activity in this group of patients. The pharmacokinetic analyses confirmed the preclinical data, in that there was extensive apparent distribution and rapid clearance of penclomedine. In these studies, no metabolites were detected in the patients' plasma.

In preclinical studies performed in dogs, doses of penclomedine associated with plasma concentrations of 6–9 µg $ml^{-1}$ were not associated with neurotoxicity, but after higher doses neurotoxicity was dose limiting (Dixit et al, 1992). Figure 1 demonstrates that plasma concentrations approaching 10 µg $ml^{-1}$ were achieved in a patient receiving 340 mg $m^{-2}$ who experienced neurotoxicity. Myelotoxicity was also recorded in these preclinical studies, but was not apparent in this clinical trial.

The incidence of cerebellar toxicity appeared to be related to drug infusion and generally resolved spontaneously after discontinuation of therapy. There were insufficient data to formally assess relationships between pharmacokinetic parameters and toxicity data. Whether prolongation of the infusion time would circumvent the neurotoxicity was to be addressed in a protocol modification. However, the NCI had also supported preclinical studies of penclomedine in the rat in collaboration with scientists at Johns Hopkins Cancer Center, Baltimore, USA. These studies specifically investigated the neurotoxicity associated with penclomedine and suggested that pathological changes were visible in the cerebellum of rats treated with penclomedine by intraperitoneal injection (single dose ≥ 150 mg $kg^{-1}$) and that these may be irreversible (O'Reilly et al, 1996*a*). Therefore, in the

 © Cancer Research Campaign 1998

**Figure 1** Plasma concentration in patient 10 after administration of penclomedine (340 mg m$^{-2}$) by i.v. infusion interest of patient safety, all clinical studies of penclomedine administered intravenously were discontinued and phase II studies are not planned in the foreseeable future.

However, much of the preclinical anti-tumour activity data accrued using penclomedine were after its oral administration. Oral administration of penclomedine was similar to i.p. administration in degree of therapeutic effect and potency for treatment of subrenal capsule xenograft of the human MX-1 mammary carcinoma. Oral doses of 300 mg kg$^{-1}$ day$^{-1}$ on days 13, 17 and 21 resulted in complete regression of all tumours for 40 days after the last treatment, when the experiment was terminated. In advanced stage s.c. human H82 small cell lung carcinoma xenografts, oral administration of 445 mg kg$^{-1}$ day$^{-1}$ penclomedine produced six out of ten complete regressions and four out of ten partial regressions (Harrison 1991).

The anti-tumour activity after oral administration of penclomedine is present, despite the apparent low oral bioavailability of the parent compound [2% in mice (Reid et al, 1992)]. Also, there is an apparent association between neurotoxicity and the plasma concentration of the parent compound. As penclomedine is the predominant chemical species found in rat brain (O'Reilly et al, 1996*b*), these data and other studies utilizing metabolites of penclomedine (O'Reilly et al, 1996*a*) suggest that the activity of the drug is related to its metabolites and the toxicity associated with the parent compound. Therefore, the clinical evaluation of penclomedine continues, but using the oral route of administration.

**Table 2** Pharmacokinetic parameters estimates derived using plasma concentrations from the initial 24 h after administration of penclomedine

| Patient | Dose (mg m$^{-2}$ day$^{-1}$) | $V_c$ (1 m$^{-2}$) | $V_p$ (1 m$^{-2}$) | $Cl_d$ (1 h m$^{-2}$) | $Cl_t$ (1 h m$^{-2}$) |
|---|---|---|---|---|---|
| 1 | 150 | 29 | 62 | 52 | 37 |
| 2 | 150 | 25 | 67 | 56 | 40 |
| 3 | 150 | 26 | 69 | 48 | 45 |
| 4 | 250 | 31 | 70 | 59 | 54 |
| 5 | 250 | 22 | 70 | 43 | 47 |
| 6 | 250 | 27 | 45 | 18 | 20 |
| 7 | 250 | 35 | 64 | 28 | 48 |
| 8 | 340 | 26 | 76 | 67 | 60 |
| 9 | 340 | 20 | 80 | 44 | 34 |
| 10 | 340 | 28 | 61 | 18 | 20 |
| Mean | | 27 | 66 | 43 | 41 |
| s.d. | | 4 | 10 | 17 | 13 |
| CV(%) | | 16 | 14 | 39 | 33 |

### ACKNOWLEDGEMENTS

The authors acknowledge the support of the Imperial Cancer Research Fund, Medical Faculty of Edinburgh University Cancer Research Award, and thank the Western General Hospitals NHS Trust. Data monitoring was carried out by the Cancer Research Campaign Phase I/II committee.

### REFERENCES

Benvenuto JA, Hittelman WN, Zwelling LA, Plunkett W, Pandita TK, Farquhar D and Newman RA (1995) Biochemical pharmacology of penclomedine (NSC-338720). *Biochem Pharmacol* **50**: 1157–1164

D'Argenio DZ and Schumitsky A (1990) *ADAPT II User's Guide*. Biomedical Simulations Resource: University of Southern California, Los Angeles, CA

Dixit R, Lopez R, Douglas T, Muellner P, Litle L, Stolz M, Arneson D, Stedham M, Smith A and Tomaszewski JE (1992) Toxicity of a five-hour intravenous infusion of penclomedine (PEN, NSC-338720) in beagle dogs. *Proc Am Assoc Cancer Res* **33**: 548

Harrison SD Jr, Plowman J, Dykes DJ, Waud WR and Griswold DP Jr (1991) Preclinical antitumour activity of penclomidine in mice: cross resistance, schedule dependence and oral activity against tumour xenografts in brain. *Cancer Res* **51**: 1979–1983

O'Reilly S, O'Hearn E, Rowinsky E, Struck R and Molliver M (1996*a*) Neuroanatomic studies of the cerebellar toxicity of penclomedine. *Proc Am Assoc Cancer Res* **37**: 374

O'Reilly S, Grochow L, Donehower R, Grossman S, Hartmen N, Roskes E, Ludeman S, Strong J, Struck R and Rowinsky E (1996*b*) Phase I, pharmacologic and preclinical studies of the novel alkylating agent penclomidine in patients with solid tumours. *Ann Oncol* **7** (suppl. 1): 99

Plowman J, Harrison SD, Dykes DJ, Paull KD, Narayanan VL, Tobol HK, Martin J and Griswold DP Jr (1989) Preclinical antitumour activity of an α-picoline derivative, penclomidine (NSC 338720) on human and murine tumours. *Cancer Res* **49**: 1909–1915

Prankerd RJ, Frank SG and Stella VJ (1989) Preliminary development and evaluation of a parenteral emulsion formulation of penclomedine (NSC 338720, 3,5-dichloro-2,4-dimethoxy-6-(trichloromethyl)-pyridine): a novel practically water insoluble cytotoxic agent. *J Paren Sci Tech* **42(3)**: 76–81

Reid JM, Mathieson DA, Benson LM, Kuffel MJ and Ames MM (1992). Murine pharmacokinetics and metabolism of penclomidine (3,5-dichloro-2,4-dimethoxy-6-(trichloromethyl)-pyridine, NSC 338720). *Cancer Res* **52**: 2830–2834

© Cancer Research Campaign 1998